# Literature Review on HIV-*Mtb* Coinfection and Stroke Risk

**DOI:** 10.3390/v18090965

**Published:** 2026-09-02

**Authors:** Jiwon Han, Apeksha Kamat, Jasmin Nguyen, Phat Nguyen, Katelyn Vu, Melissa Zhao, Vishwanath Venketaraman

**Affiliations:** College of Osteopathic Medicine of the Pacific, Western University of Health Sciences, Pomona, CA 91766, USA; jiwon.han@westernu.edu (J.H.); apeksha.kamat@westernu.edu (A.K.); jasmin.nguyen@westernu.edu (J.N.); phat.nguyen@westernu.edu (P.N.); katelyn.vu@westernu.edu (K.V.); melissa.zhao@westernu.edu (M.Z.)

**Keywords:** tuberculosis, human immunodeficiency virus, stroke, coinfection

## Abstract

Tuberculosis (TB) is a leading global cause of infectious disease. Growing evidence suggests that TB significantly increases the risk of comorbidity in HIV+ patients, particularly acute ischemic stroke (AIS). Prognosis is often poor, highlighting the need for early prevention and intervention. However, exact mechanisms linking HIV-*Mtb* coinfection and stroke remain poorly understood. A review of articles from 2016 to 2026 was conducted to study the risk of ischemic stroke and treatment strategies in HIV-*Mtb* coinfection. Both HIV and *Mtb* can increase stroke risk, but pathogenesis of the coinfection and relationship between the coinfection and stroke remain as proposals. Antiretroviral therapy (ART) has shown variable success rates; it may increase the risk of stroke or be associated with worse outcomes. Multi-drug-resistant TB (MDR-TB) can be treated with a combination of bedaquiline, linezolid, and pretomanid (BPaL). Steroids and vitamin D supplementation with ART are associated with improved outcomes. For coinfection screening, the monocyte-to-lymphocyte ratio (MLR) and a clinical scoring system for triage may prove useful for early detection, leading to prompt intervention. A clearer understanding of the association between HIV-*Mtb* coinfection and stroke is essential for developing effective prevention strategies and eliminating transmission. Advancement of current treatments will help improve long-term outcomes in HIV-*Mtb* coinfection.

## 1. Introduction

Tuberculosis (TB) is a disease caused by *Mycobacterium tuberculosis* (*Mtb*) [1]. According to the World Health Organization (WHO), an estimated 10.7 million cases of TB occurred, and 1.23 million died from TB globally in 2024; the TB incidence rate (new cases per 100,000 population per year) was 131, and the case fatality rate was 11.5%. TB is one of the top 10 causes of death worldwide and the leading cause of death from a single infectious agent. TB can be divided into pulmonary tuberculosis (PTB) and extrapulmonary tuberculosis (EPTB). PTB is the most common, while EPTB is encountered by clinicians less frequently and involves organs such as the pleura, lymph nodes, abdomen, genitourinary tract, skin, joints, bones, or meninges [2]. EPTB constitutes about 15 to 20% of all TB cases [3]. Due to its variety of presentations, EPTB often poses a great difficulty in early diagnosis [2].

Human immunodeficiency virus (HIV) is a virus which targets the host’s immune system [4]. It is transmitted sexually or from body fluids such as blood, semen, pre-seminal fluids, and vaginal fluids. For transmission to occur, the fluids must either contact the mucous membrane or be injected into the bloodstream. Some infected individuals remain asymptomatic while most show flu-like symptoms within 2 to 4 weeks after infection, with symptoms such as fever, sore throat, swollen lymph nodes, rash and night sweats. At the end of 2024, the WHO estimated around 40.8 million people living with HIV, of which 1.4 million were children and 39.4 million were those at or above the age of 15 [5]. HIV infection can be divided into 3 stages: (1) acute, (2) chronic, and (3) acquired immunodeficiency syndrome (AIDS). Stage 1, acute HIV infection, manifests as large detectable HIV present in the blood, is highly contagious, and causes flu-like symptoms. Stage 2, chronic HIV infection, occurs when HIV is still active and contagious in the host, but the host remains asymptomatic, otherwise known as clinical latency. When the host doesn’t receive HIV treatment or has severe immune system damages, the infection progresses into the final and most severe stage, AIDS, which is diagnosed with a CD4 cell count <200 cells/mL along with opportunistic infections or severe illnesses [4].

HIV and TB can interact synergistically in HIV-*Mtb* coinfection and increase mortality and poor prognosis. Countries with higher prevalences of HIV, such as those in Africa, have shown a greater association with TB transmission [6]. In 2013, 1 in 4 TB deaths were associated with HIV, and people affected with HIV are 29 times more likely to develop TB than those uninfected [7]. Global prevalence of coinfection in 2024 was 5.8% of all TB cases (around 640,000 individuals) and may have been as high as 14% [3,6]. Symptoms of HIV-*Mtb* coinfection can present as chronic fever, night sweats, and unexplained weight loss [8]. In 2016, WHO recommended HIV positive (HIV+) patients with a positive or unknown tuberculin skin test (TST) to be put on at least 6 months of preventative antiretroviral therapy (ART). For those diagnosed with active TB, antituberculosis treatment should be initiated first, then followed up with ART within the first 8 weeks of treatment [9]. ART has shown to reduce the incidence of TB as much as 50% for those receiving a year’s worth, as well as reducing symptoms and increasing longevity for those infected with HIV [9,10]. However, ART can also react poorly with coinfected individuals, leading to worsening symptoms and even death [9].

WHO defines acute ischemic stroke (AIS) as a focal neurological deficit caused by vascular occlusion, with sudden onset and symptoms lasting longer than 24 h [11]. According to the Global Burden of Disease in 2021 alone, stroke was the third leading cause of death with about 7.3 million deaths [12]. A serious complication of HIV-*Mtb* coinfection is AIS. Both pathogens on their own constitute as risk factors for AIS, which can in turn increase mortality [13]. Ortiz et al. reported 12.5% mortality and 22.5% severe disability in HIV patients due to AIS [14]. Tuberculous meningitis (TBM) occurs when *Mtb* infects the meninges and is a common form of bacterial meningitis with high morbidity and mortality. It is also the most common manifestation of EPTB involving the central nervous system (CNS). Symptoms of TBM include those related to cranial nerve involvement, headache, decreased level of consciousness, and neck stiffness [15]. Severity of TBM depends on the clinical staging and host immune system activity. TBM can present with hydrocephalus, cranial nerve palsies, dementia, and hyponatremia. Stroke is one of the most common complications from TBM, with 30% of TBM patients presenting with stroke [16]. Mortality increases for those older than 60, highlighting the importance of early diagnosis and treatment [16]. It is estimated that around 164,000 adults developed TBM globally, with 23% diagnosed with HIV [17].

Besides opportunistic infections such as HIV and TB, 84% of stroke burden in 2021 could be attributed to 23 modifiable risk factors, many of which are preventable. Of the risk factors, high body mass index (BMI), high ambient temperature, high fasting plasma glucose, diets high in sugary drinks, low physical activity, high systolic blood pressure, and lead exposure had increased disability-adjusted life years (DALYs) [12]. Although preventable, unequal access and lack of rehabilitation services result in a high stroke burden in developing countries [18].

Current understanding of the pathogenesis of HIV-*Mtb* coinfection in association with stroke is understudied. Theories on the mechanisms of HIV-*Mtb* coinfection itself are controversial with contradictory findings [19]. While there are studies highlighting stroke with each individual pathogen, there are few studies focusing on stroke in the coinfection. More knowledge about the relationship between stroke and HIV-*Mtb* coinfection is necessary for better measures of prevention, diagnosis, and treatment. The high HIV and TB incidence rates in developing countries and higher mortality rates are exacerbated by the increased risk of stroke. Early treatment is critical for improving patient prognoses and lowering mortality rates. However, because the mechanisms of coinfection are still unclear, identifying the condition at an early stage is clinically challenging. The current review aims to: highlight current insights on the pathogenesis of HIV-*Mtb* coinfection, investigate HIV-*Mtb* coinfection in association with ischemic stroke, and delve into current and upcoming diagnostic and treatment strategies that may improve long-term outcomes for HIV-*Mtb*-coinfected patients.

## 2. Materials and Methods

This narrative review summarizes the current evidence on the risk of comorbid conditions, specifically ischemic stroke, in coinfection of HIV and *Mtb* as well as treatment strategies and evaluation of long-term outcomes of the coinfection. A literature search using the PubMed database was conducted from 10 June to 19 June 2026 to determine the relevant literature published between January 2016 and June 2026. All articles older than the past decade were excluded to reflect up-to-date information. Search terms included: “HIV AND tuberculosis coinfection,” “HIV AND mycobacterium tuberculosis,” “HIV AND tuberculous meningitis AND stroke,” “tuberculous meningitis AND stroke,” “tuberculosis AND stroke,” “tuberculosis meningitis AND HIV,” “neurological disorders in HIV,” “ischemic stroke AND tuberculosis,” “stroke AND mycobacterium tuberculosis,” “risk factors AND stroke,” “stroke treatment AND antiretroviral therapy,” and “HIV and tuberculosis coinfection AND treatment.”

Original research studies, systematic reviews, narrative reviews, meta-analyses, non-randomized comparative studies, randomized controlled trials, and case studies addressing HIV-Mtb coinfection were eligible for inclusion. Surveys, interviews, and articles not directly relevant to the review objectives were excluded. Only open access articles available in English were considered. As this was a narrative rather than a systematic review, the search was not intended to identify every eligible publication, and formal assessment of study quality or risk of bias was not performed.

A total of 25 articles were initially identified, with 10 eventually selected for review. Studies were excluded from the extracted results after the initial search because they were either (a) initially thought to be relevant but upon further review beyond the abstract did not fit our objectives or (b) found to not fit our inclusion criteria such as publication date. More specifically, studies were excluded during secondary screening if they duplicated clinical endpoints already established by higher-level evidence, analyzed identical patient cohorts, or replicated known mechanistic pathways without offering novel insights. Articles were prioritized by recency and clinical relevance. Two articles, a case study by Pasticci et al. and a clinical study by Schutte, were used as references and for supporting information but were not selected as part of the primary narrative as they fell out of the January 2016–June 2026 window. The selected literature was reviewed and synthesized narratively; the findings were organized into thematic categories including the mechanisms of HIV and *Mtb* coinfection, increased risk of stroke with HIV-*Mtb* diagnosis, and treatment options for the diagnosis.

We extracted the relevant information from each article, including pertinent findings and limitations (Table 1). The review by Bell et al. was selected for discussion of the pathogenesis of HIV-*Mtb* coinfection. The review by Yang et al. was chosen for its discussion of treatment for the coinfection with ART, TB preventative therapy, and host-directed therapy. The cohort study by Lee et al. was chosen for evaluation of the increased risk of ischemic stroke in TB patients. The review article by Chutinet et al. was selected for its discussion of mechanisms of how HIV and bacterial infections together increase the risk of stroke. The meta-analysis and systematic review by Mekonen et al. were chosen for the discussion of the efficacy of the treatment for the coinfection. The randomized controlled trial study by Meintjes et al. was selected for its evaluation of the effectiveness of prophylactic treatments in HIV in the reduction in the risk of TB. The exploratory case–control study by Zimba et al. was selected for its investigation into whether ART in HIV patients increases the risk of stroke. The non-randomized comparative clinical trial study by Verma et al. was selected for the discussion on supplementation of ART with vitamin D in HIV as a protective agent against development of TB. The longitudinal cohort study conducted by Choudhary et al. was selected for the potential use of MLR in TB diagnostics and as a treatment response indicator for HIV-positive patients. Lastly, Hanifa et al.’s prospective cohort study was chosen for the discussion of a standardized clinical scoring triage system for coinfected individuals.

## 3. Results

### 3.1. Pathogenesis of HIV-Mtb Coinfection

**Figure 1:**
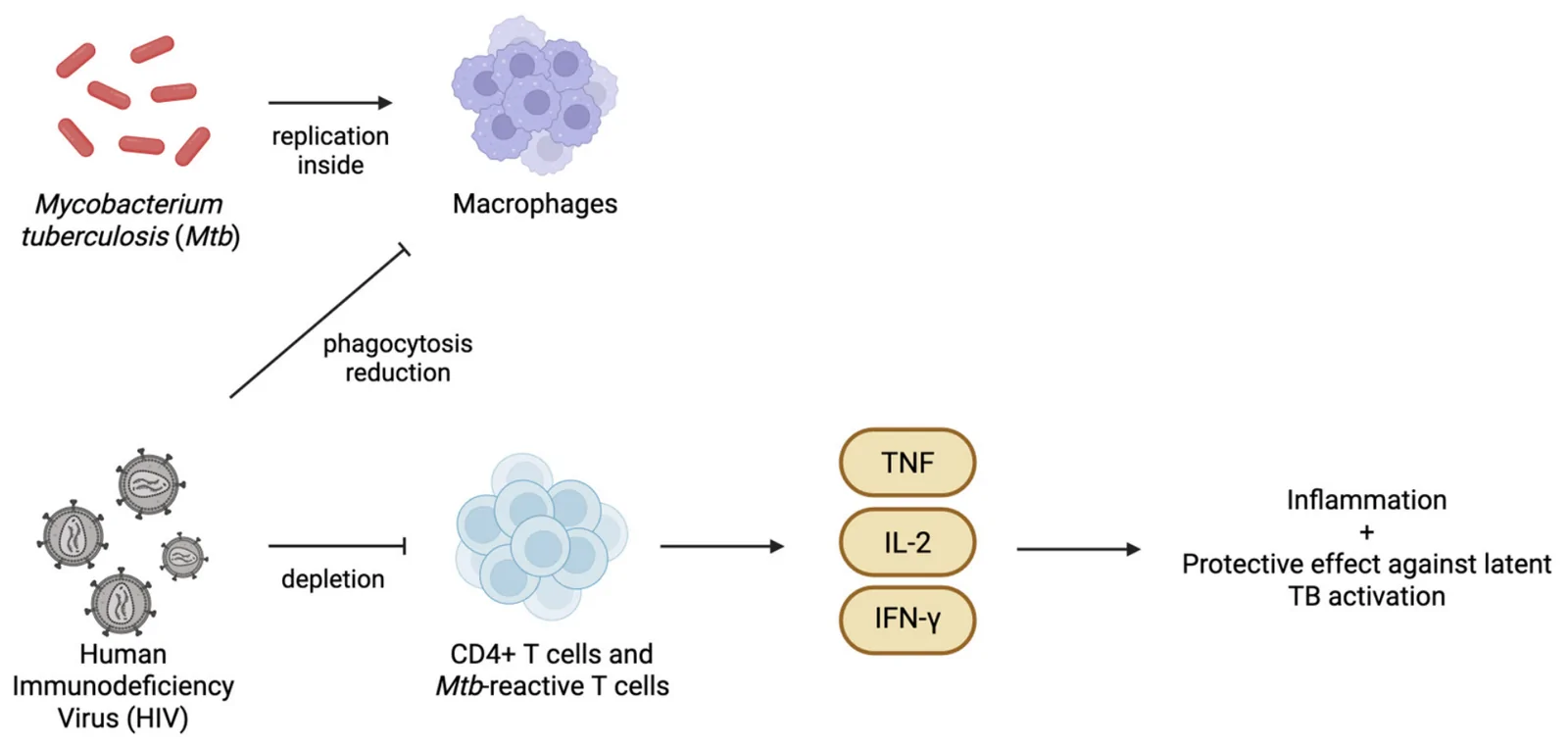
Mechanism of HIV-*Mtb* coinfection. Mtb infects and replicates in macrophages, while HIV reduces the phagocytic ability of macrophages and depletes CD4+ and *Mtb*-reactive T cells, which normally produce inflammatory cytokines, such as TNF, IL-2, and IFN-γ. In other words, HIV leads to a reduction in those inflammatory cytokines.

The infectious and physiological mechanisms of HIV-*Mtb* coinfection and stroke are unclear. The pathogenesis of HIV-*Mtb* is complex and involves multiple dysregulations. Coinfection involves the depletion of both host CD4+ T cells and *Mtb*-reactive T cells. *Mtb*-reactive T cells produce cytokines that induce inflammation, such as tumor necrosis factor (TNF), interleukin-2 (IL-2), and interferon-gamma (IFN-γ). Since these cytokines are downregulated, it is believed that their protective function decreases, which is speculated to be associated with an HIV individual’s increased risk of the conversion of latent TB to active TB [29,30]. Macrophages are also involved in the immune dysregulation of HIV-*Mtb* coinfection. *Mtb* replicates and survives by growing inside host macrophages, and in HIV infection, the macrophage’s ability to phagocytose decreases (Figure 1). Thus, we have seen that HIV-infected macrophages have an increased *Mtb* bacterial load. This then accelerates the growth of *Mtb* and increases HIV-1 replication in coinfected cultures [31,32].

Bell et al. proposed that increased HIV replication can be caused by an innate immune response to *Mtb* by macrophages producing cytokines and chemokines that recruit T cells in the host. These activated T cells allow for HIV to rapidly propagate through direct cell–cell contact and spread of the pathogen, and the cytokines promote HIV replication through the activation of transcription factors, such as nuclear factor-kB (NF-kB) and nuclear factor of activated T cells (NFAT). *Mtb* also reduces inhibition of HIV transcription through a decrease in interleukin-10 (IL-10), further promoting HIV replication [19]. Although promising, these findings are debated as other studies have reported no increased *Mtb* growth in HIV-infected macrophages [30].

### 3.2. HIV and Mtb Coinfection Impacts on Stroke Risk

Stroke mechanisms can be described as two mechanisms: direct or indirect. Direct mechanisms involve direct damage to the CNS with local inflammatory responses and arteritis resulting in ischemic stroke, while indirect mechanisms involve prolonged inflammatory responses that can result in the formation of septic emboli. The septic emboli can then result in vasculitis and induce clot formation, thus increasing stroke risk. TB involves a direct mechanism of stroke, as it induces local inflammatory responses and arteritis, while HIV involves an indirect mechanism, as chronic HIV infections result in proinflammatory states that result in vasculitis. The main mechanism behind the increase in stroke risk in TBM is the exudation of the leptomeningeal layer at the base of the brain, which causes inflammation of the vessels surrounding the circle of Willis [22,33]. Additionally, per Chutinet et al., thickening of the intima layer and vasospasm results in hemodynamic hypoperfusion, which can lead to stroke. In an acute infection, protein S levels are decreased, while factor VIII and plasminogen activator-inhibitor-1 are activated.

Since those with HIV are at a higher risk of TB activation, their risk of stroke may also increase, though the mechanism is not clear. In a cohort study conducted on the Korean population, 72,863 cases of confirmed TB diagnosis from the years 2010 to 2017 were obtained, and 72,863 non-TB cases used to match the data were analyzed. In total, 1.3% of the patients with TB developed ischemic stroke, and after adjusting for risk factors, population demographics, and comorbidities, Lee et al. found that TB-diagnosed patients had a 1.22 (95% CI, 1.10–1.36) times higher risk of ischemic stroke than their matched cases. TB-diagnosed individuals also had an increased risk of stroke overall compared to non-TB cases when common risk factors were considered, except for patients who were 20–29 years old and those with congestive heart failure [21].

With HIV infection, stroke is already a known risk. Prolonged use of highly active antiretroviral therapy (HAART) medications can induce stroke, and 50% of strokes in HIV patients on HAART were caused by large vessel atherosclerosis. Patients with HIV infection who have a high stroke risk are associated with a high viral load and low CD4 counts [22,33].

As mentioned earlier, patients with HIV infection can be at higher risk for TB activation. However, the direct impacts of stroke risk with coinfection of HIV and TB remain unclear.

### 3.3. TB Diagnosis and Management in HIV+

Traditional treatment for HIV has been delayed antiretroviral therapy (ART) due to cost, compliance, and drug side effects [20]. However, recent studies have shown that initiating early ART can increase the CD4 T cell count [34]. This in turn is beneficial for people with HIV-*Mtb* coinfection since it allows for a more rapid immune recovery and prevents further risks of the coinfection [20].

Multi-drug-resistant TB (MDR-TB) is a result of selection of resistance *Mtb* during first-line TB treatment of rifampicin and isoniazid [35]. A single-arm, open-label study in South Africa called Nix-TB was conducted with 109 participants. Participants were 14 years old or older and were diagnosed with either extensively drug-resistant TB (XDR-TB) or MDR-TB [36]. The overall results showed that a combination of bedaquiline, linezolid, and pretomanid (BPaL) demonstrated favorable outcomes for 98 participants (90%). A total of 56 out of those 109 participants (51%) were HIV positive; 38 out of 109 participants (34%) were diagnosed with MDR-TB, and 35 out of those 38 (92%) participants demonstrated positive outcomes [36]. This study suggested that BPaL is a safe and effective treatment for MDR-TB even in people who are coinfected with HIV.

In a meta-analysis and systematic review by Mekonen et al., 34 studies were obtained to determine the effectiveness of TB treatment for coinfected patients in Ethiopia. Successful treatment was defined as patients who were cured or those who completed their treatment. Out of 7909 patients undergoing treatment, 69.94% had successful treatment. Mekonen et al.’s findings differ from WHO’s, which reported an 85% success rate in 2020. Success rates also differed from region to region. In the Amhara region, success rates were 67.9%, while regions like Harari reported success rates up to 82.5%. The pooled cure rate among studies was 19.29%, and the odds of an unsuccessful TB treatment were 2.65 (95% CI, 2.1–3.3) times higher for the coinfected than HIV-negative patients [23].

Furthermore, ART may also increase the risk of stroke. In a two-year prospective case–control study from Zambia, 205 participants that experienced a stroke on ART and 410 controls who were HIV positive but stroke-free were analyzed. Tenofovir disoproxil fumarate (TDF) is a nucleoside reverse transcriptase inhibitor (NRTI) that can be included in an ART regimen. TDF had an adjusted odds ratio of 85.3 ((95% CI, 5.3–1380.7), *p* = 0.02) for increased risk of stroke. The length of ART also may increase the risk of stroke. A total of 200 of the 205 case participants used ART for more than a year. Of these 200, 174 cases (87%) presented with stroke [25].

Although the current treatment of coinfected individuals commonly involves ART, it can sometimes worsen outcomes or activate latent tuberculosis infection (LTBI). Immune reconstitution inflammatory syndrome (IRIS) is associated with recovery of Th1 immunity but hyperactive Th2 responses [29]. A potential new treatment modality includes the use of steroids to decrease the risk of IRIS in coinfected individuals. In a randomized, double-blind, placebo-controlled trial conducted by Meintjes et al., 240 patients infected with HIV who had not yet initiated ART and were receiving antituberculosis treatment were chosen. Prednisone was given in conjunction with the initiation of ART to the experimental group. Those who took prednisone had a 30% lower incidence of TB-associated IRIS than placebo (relative risk 0.70, (95% CI, 85–96.2)). Fewer patients in the experimental group met at least one of the International Network for the Study of HIV-associated IRIS (INSHI) criteria (relative risk 0.57), and the experimental group had more patients with better outcomes than the placebo group. The prednisone group had fewer hospitalizations and less interruption of ART treatment due to adverse events, although the data was not statistically significant [24].

In addition, vitamin D can also be considered as a supplement to treatment, as it has been shown to inhibit HIV replication in macrophages [37]. A non-randomized, comparative clinical trial conducted by Verma et al. was conducted to analyze the effects of vitamin D supplementation on CD4 count in HIV+ children and adolescents in India. A total of 50 participants on ART were split into three different groups: (1) vitamin D sufficient, (2) vitamin D insufficient, and (3) vitamin D deficient. From the beginning of the study to 4 months post-enrollment, CD4 count increased 1.2 times more in the vitamin D-deficient group (*p* < 0.001). The mean increase in CD4 count over the 4 months in the deficient group was 18.4%. There was no significant change in CD4 counts for the vitamin D-sufficient (*p* = 0.168) and insufficient groups (*p* = 0.791) [26]. These results suggest that vitamin D supplementation in conjunction with ART may be beneficial to vitamin D-deficient patients.

For HIV+ individuals, the monocyte-to-lymphocyte ratio (MLR) may be used as an accessible TB diagnostic biomarker and indicator for treatment response. In a longitudinal cohort randomized clinical trial study, 160 children with HIV were split into three groups: confirmed, unconfirmed, or unlikely TB diagnosis. The median MLR for those with a confirmed TB diagnosis (0.407) was higher than those with an unconfirmed (0.207, *p* < 0.01) or unlikely TB diagnosis (0.391, *p* = 0.01). When comparing the unlikely TB diagnosis group with the confirmed TB group, there was a significant association between TB status and MLR (*p* = 0.01, no confidence interval reported). An optimal MLR cutoff value of 0.378 identified confirmed TB patients with a sensitivity of 77%, specificity of 78%, positive predictive value (PPV) of 24%, and a negative predictive value (NPV) of 97%. After 24 weeks of anti-TB treatment, the median MLR decreased in children with confirmed TB (*p* = 0.01) and reached similar levels to those with unlikely TB diagnosis after 12 weeks of treatment. Unconfirmed TB and unlikely TB patients showed no significant difference in the median MLR during any treatment interval [27].

Furthermore, the use of a standardized clinical scoring system for coinfected patients may be a beneficial screening tool for early diagnostics and triage. A prospective cohort study conducted on 1048 patients by Hanifa et al. used data from “Xpert for people attending HIV/AIDS care: or review?” (XPHACTOR), which involved a questionnaire incorporating the WHO TB screening, CD4 count, and BMI, among other predictors. Patients who met the high-priority criteria through the XPHACTOR algorithm were sent for an immediate spot sputum test for Xpert MTB/RIF analysis. Patients with a clinical score of ≥3 were chosen as priority for TB investigation, with a sensitivity of 91.8% (95% CI, 85–96.2) and a specificity of 34.3% (95% CI, 31.3–37.5). Although there was low specificity, sensitivity was prioritized to avoid missing potential TB cases. Using this score, 32% of unnecessary tests would be avoided, at the cost of missing 3% of patients with confirmed TB. Those who were WHO TB screening negative or had a clinical score lower than 3 had an overall risk of 1% [28].

## 4. Discussion

### 4.1. Pathogenesis of HIV-Mtb Coinfection and Stroke Risks in Coinfection

There are multiple hypotheses on the pathogenesis of HIV-Mtb coinfection that are still debated. HIV and Mtb are both known to increase stroke risk, but it is challenging to pinpoint whether stroke from coinfection was caused by HIV infection, TB, or a combination of the two pathogens. TB and HIV alone already increase stroke risk through mechanisms that either indirectly or directly impact vascular health, with some data suggesting that TBM patients with HIV infection had more brain infarcts and dilated ventricles [38]. Coinfection also involves a complex interaction of cytokines and macrophages that may explain how Mtb can increase HIV transcription, but other studies have refuted the in-creased HIV replication altogether [19,30]. For instance, one case report published in 2013 highlights the difficulty of identifying a clear mechanism explaining coinfection. This stroke case involved a 45-year-old Caucasian female with HIV infection and diagnosis of TBM, presenting with a 1-week history of fever and 2-day history of cough [39]. A probable diagnosis was made of TBM after examination of CSF fluid and a culture of respiratory secretions showed *Mtb*. The patient had a right-sided stroke 2 days later. After the patient was admitted to their infectious disease clinic, she was diagnosed with TBM complicated with stroke after MRI showed a recent ischemic lesion and absence of flow through left middle cerebral artery [39]. In this case, there were already several existing risk factors for stroke such as a previous drug habit and smoking, along with the HIV infection and the new TB diagnosis. All of these could have played a role in the development of vascular pathologies and ultimately stroke. The most likely diagnosis in this case was vasculopathy secondary to TB, given that the patient had a coinfection of HIV with a low CD4+ T lymphocyte count and brain imaging consistent with TBM [39].

Current research on stroke risk and coinfection relies on case reports and self-reports of stroke or produces findings that may not be most applicable to individuals who are most affected by TB. Many patients who suffer from HIV-Mtb coinfection reside in low-income and developing countries, which makes conducting studies on these vulnerable populations more challenging due to resource limitations. Further research is needed to better understand the vascular pathophysiology of coinfection and its impact on stroke risks. For a clearer understanding, potential future research endeavors should compare the vascular health and any vascular changes in patients with both HIV and TB to the following groups: patients only infected with HIV, patients only infected with *Mtb*, and patients with neither HIV nor TB. This information is important when applied to HIV individuals, as their risk for reactivation of LTBI is increased, and therefore the risk of stroke is increased. Understanding the pathogenesis pathways will improve patient outcomes as well as prevent worsening outcomes.

### 4.2. Current Treatment Advancements for HIV-Mtb

The standard treatment for coinfected individuals is far from perfect. The WHO’s guidelines for managing coinfection have remained the standard treatment, but studies have demonstrated that ART can fail and even cause additional harm to those who take it [23,25]. The odds of an unsuccessful treatment were higher for coinfected individuals, and certain ART drugs such as TDF may increase the risk of stroke and IRIS [23,25,29]. This stresses the need for further investigation into different treatment avenues. Treatment adherence is considered crucial in preventing HIV from replicating in the body, and improving access to treatment can assist with treatment compliance.

The first line of treatment of TB is rifampicin and isoniazid. However, if this first line of treatment fails, as in the case of MDR-TB, the BPaL regimen can be used and has shown favorable outcomes for those diagnosed with MDR-TB as well as those who are HIV+ [36]. The WHO recommended that bedaquiline and linezolid, the two components of the BPaL regimen, be used as the first line of treatment for MDR-TB over an 18-month course of therapy [40]. Data from the Nix-TB trial reported that prior to the use of bedaquiline and linezolid, the cure rates were below 20% for XDR-TB. The cure rates improved to 66% when using bedaquiline and linezolid [36]. One of the limitations of the study was that it was only conducted in South Africa; therefore the data could not be generalized to other susceptible populations [36]. The high dose of linezolid also caused 80% of the participants to report peripheral neuropathy, and 50% had evidence of hematological toxicity [36]. However, the toxicity of linezolid can be controlled by lowering its dose without affecting its effectiveness in treating TB [20].

In addition, supplements to current standard treatment for coinfected individuals show potential to improve patient outcomes. Steroids show promise to combat complications that occur from ART, and vitamin D supplementation with ART has been shown to increase CD4 count in vitamin D-deficient individuals [24,26]. Prednisone is a widely available and affordable drug, which is advantageous for under-resourced areas that have high prevalence of HIV-*Mtb* coinfection with low economic status patients. As discussed, low CD4 count was important for its potential role in HIV-*Mtb* pathogenesis and HIV infection stroke risk [22,29,30,33]. Since vitamin D was found to increase CD4 cell counts in HIV patients, it has potential to aid in the restoration of immune function and possibly reduce the risk of stroke. These findings are beneficial to coinfected individuals who are already at risk of other health complications, such as stroke. More research is necessary to investigate whether an increased dose of prednisone provides greater benefits and what other steroids show similar effects or potentially even better effects. Screening for HIV-*Mtb* coinfection should assess for vitamin D deficiency, and supplementation with ART should be considered for those with vitamin D deficiency. A future study should also observe sun exposure in patients, which may give more insight into vitamin D levels for vitamin D supplement treatment efficiency.

Genome sequencing may also help us better understand how *Mtb* affects HIV and vice versa [19]. If a target in the coinfection relationship can be identified, it can help modify current treatments to be more effective and successful. Efforts to achieve a better understanding of the synergistic relationship between HIV and *Mtb* must continue to prevent the reactivation of LTBI as well as complications that result from the coinfection. In addition, incorporating lifestyle modifications such as quitting smoking, as well as proper maintenance of metabolic disease, should be implemented with treatment to decrease the risk of stroke.

Current studies on treatment modalities for coinfection struggle with generalizability, time constraints, small sample sizes, or success rates from treatment that may be inaccurate due to difficulties initiating treatment and diagnosing coinfected patients in settings with weak healthcare systems that lack resources. In addition, many of these studies do not evaluate patients for risk factors of stroke, such as diabetes and hyperlipidemia [25]. Although some drugs in ART regimens such as TDF may cause stroke, other ART drugs and combinations that participants take have not been assessed on how they may impact stroke risk. More research needs to be done on the association of stroke with other drugs in ART regimens, as well as ART combinations with current and upcoming treatments [25]. Evaluations for treatments should adjust for common risk factors of stroke to identify independent associations for the increased risk of stroke associated with ART drugs and combinations. Identifying potential stroke-inducing medications and improving treatment adherence are crucial to prevent further complications for coinfected individuals.

### 4.3. Diagnostic Advancements for HIV-Mtb Coinfection

Screening and diagnostics are crucial to detecting HIV-*Mtb* coinfection early and for preventing future transmission. MLR’s ability to identify potential confirmed TB cases in HIV patients, as well as indicate treatment response, shows promise as a cheap diagnostic alternative to current gold standards such as Xpert MTB/RIF [27]. Electricity problems, cartridge availability, high cost, transportation issues, and the lack of education and testing training have led to difficulties implementing Xpert MTB/RIF in low-resource countries [41]. The addition of a standardized clinical score to screening may also benefit resource management and early detection of HIV-*Mtb* coinfection. There is currently no standardized system that prioritizes HIV-*Mtb* patients, but XPHACTOR may provide clinical professionals with better insights into which patients present with more prominent TB findings than others [28]. Having a prioritization system is useful for hospitals and clinics that need to triage resources that are less available or expensive. It also decreases the possibility of wasting assets on a patient that may not be coinfected.

Future research on these diagnostic techniques should be tested on larger sample sizes across different demographic backgrounds and locations to ensure effectiveness, high sensitivity, and specificity. XPHACTOR also utilizes CD4 count in its clinical score, which may not be readily monitored, so other scoring models that do not use CD4 count and are as successful as XPHACTOR should be considered. These techniques should ideally be low-cost, easy to learn, and widely accessible. Improving diagnostics will improve transmission prevention and earlier initiation of treatment, which will in turn reduce complications and mortality associated with coinfection.

## 5. Conclusions

TB is a leading global cause of infectious disease and death among HIV patients. HIV-Mtb coinfection significantly accelerates disease progression through inflammatory cytokine-mediated pathways, though the exact mechanisms remain debated. Both pathogens independently and synergistically elevate ischemic stroke risk by inducing a pro-inflammatory state that promotes clot formation which may be further compounded by traditional vascular factors.

Optimal clinical management relies on carefully timed ART initiation following anti-TB treatment to minimize IRIS and vascular complications. Emerging strategies, including BPaL regimens for MDR-TB, adjunct steroids and vitamin D supplementation, and predictive tools like MLR, show promise in improving diagnostic speed and therapeutic efficacy. Ultimately, reducing the associated stroke risk and improving long-term outcomes in coinfected populations require early screening and detection, treatment adherence, and targeted research into the mechanistic details of HIV-Mtb coinfection.

## Figures and Tables

**Table 1:** Summary of selected studies on HIV-*Mtb* coinfection, stroke risk, and management. Overview of included publications stratified by author, publication year, country, study design, target population, and summary of key statistical or narrative findings regarding coinfection mechanisms, ischemic stroke risk factors, and therapeutic interventions.

Author	Country	Study Design	Population (N)	Significant Statistical Findings	Key Findings	Limitations
Bell et al. [19]	N/A	Review	N/A	N/A	Macrophage respond to Mtb by producing cytokines and chemokines that may cause increased HIV replication	Narrative Review
Yang et al. [20]	N/A	Review	N/A	N/A	BPaL is a potentially safe and effective treatment for MDR-TB and HIV positive patients	Narrative Review
Lee et al. [21]	Korea	Cohort Study	72,863 final TB survivors and 72,863 matched non-TB cases	aHR 1.22 [95% CI, 1.10–1.36]	Tuberculosis survivors had a higher risk of ischemic stroke than their matched non-tuberculosis cases	Possible recall bias, generalizability concerns
Chutinet et al. [22]	N/A	Review	N/A	N/A	Both pathogens, Mtb and HIV individually as well as together can increase the risk of stroke	N/A
Mekonen et al. [23]	Ethiopia	Meta Analysis and Systematic Review	7909	OR 2.65 [95% CI: 2.1 to 3.3]	Unsuccessful TB treatment were higher for coinfected rather than HIV negative patients	Generalizability concerns
Meintjes et al. [24]	Zambia	Randomized controlled trial study	24,024	RR 0.70 [95% CI, 0.51–0.96], *p* = 0.03	Prednisone may decrease risk of IRIS in coinfected individuals	Generalizability concerns
Zimba et al. [25]	Zambia	Exploratory case-control study	205 cases and 410 controls	aOR 85.3 [95% CI, 5.3–1380.7], *p* = 0.002	TDF is associated with increased odds of stroke	Generalizability concerns
Verma et al. [26]	North India	Non-Randomized comparative Clinical Trial	50	1.2-fold rise in CD4 count in vitamin D deficient group, *p* < 0.001	Vitamin D supplementation in conjunction with ART increases CD4 count for HIV positive patients	Time constraint and small sample size
Choudhary et al. [27]	Kenya	Longitudinal Cohort Study	160	Median MLR decreased in confirmed TB children taking anti-TB treatment, *p* = 0.01	MLR can be used as an indicator for treatment response	Generalizability concerns and small sample size
Hanifa et al. [28]	South Africa	Prospective cohort study	1048	91.8% sensitivity [95% CI, 85–96.2], 34.3% specificity [95% CI, 31.3–37.5]	XPHACTOR standardized clinical scoring can be used to triage patient testing and screen coinfected patients	Generalizability concerns

Key: aOR, adjusted odds ratio; CI, confidence interval; aHR, adjusted hazard ratio; OR, odds ratio; RR, relative risk; N/A, not available.

## Data Availability

No new data was created in the production of this literature review. Data presented in this review was derived from the following public domains: https://pubmed.ncbi.nlm.nih.gov/ (accessed on 10–19 June 2026).

## Abbreviations

The following abbreviations are used in this manuscript:

AIS	Acute ischemic stroke
ART	Antiretroviral therapy
BPaL	Bedaquiline, linezolid, and pretomanid
BMI	Body mass index
DALY	Disability-adjusted life year
CNS	Central nervous system
XDR-TB	Extensively drug-resistant tuberculosis
EPTB	Extrapulmonary tuberculosis
IRIS	Immune reconstitution inflammatory syndrome
IFN-γ	Interferon-gamma
IL-2	Interleukin-2
IL-10	Interleukin-10
INSHI	International Network for the Study of HIV-associated IRIS
HAART	Highly active antiretroviral therapy
HIV	Human immunodeficiency virus
HIV+	HIV positive
LTBI	Latent tuberculosis infection
MLR	Monocyte-to-lymphocyte ratio
MDR-TB	Multi-drug-resistant tuberculosis
*Mtb*	*Mycobacterium tuberculosis*
NPV	Negative predictive value
NF-kB	Nuclear factor-kB
NFAT	Nuclear factor of activated T cells
NRTI	Nucleoside reverse transcriptase inhibitor
PPV	Positive predictive value
PTB	Pulmonary tuberculosis
TDF	Tenofovir disoproxil fumarate
TST	Tuberculin skin test
TB	Tuberculosis
TBM	Tuberculosis meningitis
TNF	Tumor necrosis factor
WHO	World Health Organization
XPHACTOR	“Xpert for people attending HIV/AIDS care: or review?”
